# Nursing staff’s responses to thematic content of patients’ expressed worries: observing communication in home care visits

**DOI:** 10.1186/s12913-018-3390-5

**Published:** 2018-08-03

**Authors:** Linda Hafskjold, Vibeke Sundling, Hilde Eide

**Affiliations:** 1 Department of optometry, radiography and lighting design, Faculty of Health and Social Sciences, University of South-Eastern Norway, Drammen, Norway; 2 National Centre for Optics, Vision and Eye Care, Faculty of Health and Social Sciences, University of South-Eastern Norway, Kongsberg, Norway; 3 Science Centre Health and Technology, Faculty of Health and Social Sciences, University of South-Eastern Norway, Drammen, Norway; 4 Faculty of Health and Social Sciences, University of South-Eastern Norway, PoBox 7053, N-3007 Drammen, Norway

**Keywords:** Communication, Emotions, Expressed emotion, Home care services, Older adult, Nursing staff, Observational study, Person-centred communication

## Abstract

**Background:**

The aim of the study was to explore the thematic content of older persons’ expressed worries in home care visits, and how nursing staff respond to different thematic contents.

**Methods:**

The study had a descriptive, observational design, including 195 audio-recorded Norwegian home care visits with 33 nursing staff and 48 older persons. In all, 638 patient cues/concerns (worries) and subsequent nursing staff’s responses were identified using Verona Coding Definitions of Emotional Sequences. A novel thematic coding scheme was used to label the thematic content of the cues/concerns. The nursing staff’s responses were grouped based on communicative function as emotion-focused, content-focused or ignoring/blocking the cue/concern. Group difference was analysed using Pearson’s chi-squared test, Fisher’s exact test, and adjusted residuals.

**Results:**

The theme of worries was associated with elicitation of the cue/concern, either elicited by the nursing staff or spontaneously expressed by the patient (Chi-square, *p*< 0.001). “Ageing and bodily impairment” was the most common theme (66%) and was equally elicited by patients and nursing staff. Worries about “Relationships with others” (9%), “Health care-related issues” (15%) and “Life narratives and value issues” (9%) were mainly elicited by nursing staff. The nursing staff response was associated with the theme of worries (*p*˂0.001). For the sub-themes of “Ageing and bodily impairment”, *Coping with existential challenges* received more frequently emotion-focused responses (adjusted residuals: 3.2) and *Expression of pain felt in the moment* were more frequently ignored/blocked (adjusted residuals: 4.0, Fisher’s exact test, *p*< 0.001). For the sub-themes of “Relationships with others”, *Being a burden* more frequently received a content-focused response (adjusted residuals: 2.8), while *Losing social ties* more frequently received an emotion-focused response (adjusted residuals: 3.1, Fisher’s exact test, *p* = 0.009).

**Conclusion:**

“Ageing and bodily impairment” was the most common theme and more frequently elicited by the older persons than other themes. Emotionally focused nursing staff responses were most common when addressing existential challenges and fear of losing social ties. Whereas nursing staff showed a tendency to ignore patients’ spontaneous expressions of pain. Further research should explore the influence of nursing staff’s responses on quality of care and patient satisfaction.

## Introduction

### Background

How care providers communicate with older persons can have a significant impact on older persons’ perception of well-being and quality of life, as well as cognitive and functional abilities [1]. Across healthcare settings, the provision of high-quality healthcare depends on the care provider’s ability to notice and respond to patients’ worries in order to meet patients’ care needs, to achieve care outcomes, and to ensure a trusting care relationship [2–7]. Older persons describe high quality care as related to being recognised as a person with a unique history, experiences and competences [8–10], that is an underpinning principle of person-centred care [11].

To achive the goals of high-quality healthcare, nursing staff need to work together with care recipients, and involve them as partners in all steps of the care delivery, that is a hallmark of a person-centred healthcare [12]. Norwegian policy for older people promote active ageing, providing older people to live at home as long as possible [13, 14]. Person-centred healthcare, perserving autonomy and independence, despite illness and functional impariment, with respect to the older person’s preferences and values, is an important aspect of the policy. This is in accordance with description of person-centred healthcare tailored towards older people’s needs [15] and the World Health Organization strategic objectives to ensure sustainable healthcare systems [16]. To ensure effective care delivery that supports the older person to stay self-reliant and in control over their life, nursing staff need to be aware of how older persons express deterioration of health and well-being [17]. Current research describes the importance of addressing emotional needs of older persons living at home, but few studies have analysed how nursing staff respond to expressed emotional needs during home care visits [18]. Moreover, the research builds mainly on interviews [19–21] and surveys [22–24].

This paper is based on an international research project, the COMHOME study, which focus on how older persons communicate with their care providers [25]. Qualitative studies from COMHOME show that communication in home care mainly is instrumental, focusing on the task at hand [26, 27], similar to reported findings 20 years ago [28]. A study, from Sweden and Denmark found that in home help visits, task completion aims at being “good enough” rather than “brilliant” and that older persons rarely dispute the home help provider’s assessment of the task performed [29]. This could be a reason why communication challenges between older persons and nursing staff seem to occur when the conversation takes an unexpected turn away from the instrumental care task, like when the older persons express concerns, have contradicting views, or raise existential issues, as identified in the COMHOME study [26]. Other studies underline the importance of establishing a professional *friendship* between nursing staff and the older person receiving home care, to elicit the patient perspective, and ensure respect in all steps of care delivery, and to foster a supportive care environment [19]. A well-functioning relationship between the patient and the home care nurse builds on trust, when trust is achieved, the patient’s sense of security is increased and loneliness is relieved, especially for persons lacking social support [30]. Moreover, the face-to-face meetings in the home of the patients provide unique information and understanding needed to provide good and safe care [31]. A good patient-nurse encounter in home care was characterised by security, presence, time, respect and seeing the person. Moreover, ensuring that home care nursing staff is able to prioritise time for psychosocial talk, allowing the patient to share emotional struggle, and not only instrumental assistance has been emphasised as an important health-promoting measure for home care services, especially for older persons living with multimorbidity [32].

To identify and quantitatively analyse patient expressions of worries and nursing staff’s subsequent responses during emotional talk in observational studies, the “Verona Coding Definitions of Emotional Sequences” (VR-CoDES) has been developed [33, 34]. The VR-CoDES defines a concern as “*a clear and unambiguous expression of an unpleasant current or recent emotion where the emotion is explicitly verbalized*” and a cue as the patient’s “*verbal or non-verbal hint which suggests an underlying unpleasant emotion but lacks clarity*” [33]. The COMHOME study has shown that older persons communicate their worries mainly as cues and not as explicitly stated concerns during home care visits [18, 26, 35]. This is also seen in studies in other care settings [36–38], however, these studies do not show what the worries are about. Patients in hospices and primary care are shown to be selective in their communication with nurses, often restricting the worries to physical symptoms [39, 40], but there is no knowledge about the issues raised by the patients in home care.

The COMHOME study has shown that nursing staff more often open up for the patient to elaborate about the worries when nursing staff themselves elicit the expression of worry, or when the patient uses verbal or non-verbal expressions clearly pointing to an unpleasant emotion, making the affective content more clear [41]. Similar response-patterns are also identified in other care settings [42, 43]. In primary care, robust empirical evidence support that nurses’ affective communication is related to both patient and nursing staff satisfaction, and affect is more clearly communicated through speech and vocal tone than through non-verbal channels [7]. To our knowledge no recent studies have investigated the immediate nursing staff response to patient expressed worries in home care visits.

Doctors and nurses respond differently to expressions of worries [43, 44]. Nurses more often allow emotions to be disclosed [43]. This may reflect role expectations and healthcare context, as well as how doctors and nurses consider the importance of exploring emotional issues [5, 45–47]. However, patient satisfaction increases when worries are acknowledged and explored [48], and there is a lack of knowledge about how care providers adjust their response according to the thematic content of worries.

There is a need to better understand what happens and how the care provider respond to different themes of worries. Studies indicate that care providers find it difficult to communicate with older persons about existential worries, mental health issues, and issues having an unpleasant emotional value to the person [26, 32, 49]. It is possible that thematic content in emotional expressions perceived as difficult to address by nursing staff, receive less space for discussion and consequently reduce the older persons’ opportunity to receive help to resolve and cope with particular worries. This could have an impact on the older persons ability to continue to live a good life at home [32].

Being able to attend to older persons’ needs for physical, psychological, social, and spiritual support are described as crucial to help older persons maintain a positive spirit in late life and to cope with potential challenges arising from reduced health or other issues potentially threatening their well-being [50]. Therefore, describing nursing staff’s responses related to thematic content of patients’ expressed worries may provide an important insight into the verbal disclosure of concerns in home care visits [18].

The aim of this paper is to explore characteristics of the thematic content of older persons’ expressed worries in home care visits and nursing staff response to different thematic contents.

## Methods

### Design, setting and sample

The study had a descriptive, observational design [51, 52]. The data was collected as part of the COMHOME study [25].

#### Setting and sample

Between December 2013 and May 2014, audio recordings of 195 home care visits were collected at four Norwegian home care units covering both rural and urban populations. The audio recordings captured communication between older patients (≥65 years) and registered nurses (RN) or nurse assistants (NA) during the home care visit.

Nursing staff and patients were recruited based on eligibility criteria and provided informed consent to participate. The **nursing staff criteria** was being an RN or a NA having a permanent position and working at the time of data collection. In Norway, RNs and NAs have formal education at bachelor degree and upper secondary school level, respectively. A key management person at each home care unit ensured that all eligible nursing staff received written information about the study. In addition, two members of the research team provided oral information about the study on two occasions at each home care unit. Nursing staff who met the inclusion criteria gave informed consent to participate to the key management person.

The **patient criteria** was to receive home care during period of data collection and being able to provide informed consent. Effort was made to ensure variation in characteristics of nursing staff (gender, work experience), patients (age and care needs) and visits (e.g. time of day, type of assignments). Nursing staff, well-known to the patients, provided eligible patients with oral and written information. All nursing staff participating in the recruitment process received instructions for recruitment by the research team. Emphasis was put on the importance of checking that the information was correctly understood, encouraging questions, and emphasising that declining to participate or withdraw from the study was unproblematic and of no consequences to the care provided. After a minimum of 24 h, a written informed consent was collected from the patient. To ensure that all patients were confident in their decision to participate, the key management person talked to each patient after the patient had taken part in the study. Three patients declined further participation, none wanted collected data to be deleted.

Table 1 provides an overview of the nursing staff, the older patients, and the characteristics of the home care visits.

**Table 1:** Overview of sample

Nursing staff (*n* = 33)	
Registered Nurses/Nurse Assistants (n)	16/17
Females/Males (n)	27/6
Mean age (SD); age range^a^	42 (±10); 23–59
Mean years of work experience (SD); range work experience^b^	17 (±10); 1–31
Older persons (*n* = 48)	
Females/Males	36/12
Mean age (SD); age range	84 (±8); 65–94
Mean ADL^c^ (SD); range ADL	2,1 (±0,7); 0–3,7
Mean hours of care per week (SD); range hours of care per week	5 (±5): 0,3-21,5
Visits (*n* = 195)	
Mean length of visits in minutes (SD); range length of visit	17 (±14); 1–72
Number of visits with Registered Nurses/Nurse Assistants	98/98

^a^Missing data on 2 registered nurses and 2 nurse assistants

^b^Missing data on 1 nurse assistant

^c^Activity of Daily Living scores: the level of assistance needed to perform a range of daily tasks (0 = no assistance needed, 5 = full assistance needed) [95]

One workweek was dedicated for each home care unit to complete data collection. This ensured a minimum of interruption and inconvenience for participating nursing staff, other nursing staff and patients. A digital audio-recorder captured the communication as it naturally unfolded during the entire visit. All nursing staff received instructions on how to operate the digital audio-recorder, excluding the need for the researchers to accompany the nursing staff on their visits. The audio-recorder did not interfere with the nursing staff’s ability to assist the patient. Five nurses and five nurse assistants were set as the desired minimum of nursing staff to be recruited based on feasibility and reported numbers to ensure a reliable sample [7, 47].

The visits covered a range of care assignments such as delivering medication, helping with compression stockings and getting dressed, wound care, attending to personal hygiene, preparing meals, and managing assistive technology. The number of care assignments varied from single tasks to multiple tasks within the same visit. All nursing staff encountered at least three different patients, but could also encounter the same older person in multiple visits. The older person could encounter different nursing staff in multiple visits. Level of familiarity between nursing staff and the individual patient varied, but all nursing staff had access to individual record notes, list of medications and care assignments through the digital patient management system.

### Thematic coding of patients’ expressions of worry

The VR-CoDES identified moments when the patient shared his/her worries (cues or concerns) with the nursing staff, allowing the dyad of emotional communication to be analysed. Patients’ expression of worries were identified and coded according to VR-CoDES [33], capturing moments where patients raise troubling issues which they want the care providers to address [53].

To code the thematic content of the identifies worries, we developed a coding scheme based on the four over-arching themes derived from an in-depth inductive content analysis of worries expressed in home care: 1) Relationships with others, 2) Health care-related issues, 3) Ageing and bodily impairment, and 4) Life narratives and value issues, and their subcategories [18].

The first theme **Relationships with others** includes three sub-themes: 1) worries about being a burden, but still needing help from friends, family and nursing staff, 2) worries about losing self-government triggered by how practical assistance was practiced or offered, and 3) worries about losing social ties, risks of losing attachment to significant others.

The second theme **Health care-related issues** includes two sub-themes, expressions about how: 1) the care exacerbates the problem and 2) the help is being unhelpful; described as inefficient, disappointing, too late, or not helping at all.

The third theme **Aging and bodily impairment** include one sub-theme; existential challenges, capturing worries about coping with life, pondering over death, or lack of hope for the future. In addition, exclamation of pain, representing bodily sensations or experience caused by aging or some bodily impairment, is included when it appear as the moments of pain limited the older person’s ability to manage and cope in the situation. Whereas expressions of pain linked to the care procedure was assigned to the appropriate sub-theme for “Health care-related issues”.

The forth theme **Life narratives and value issues** defines emotional narratives or reflections on value issues.

**Fig. 1:**
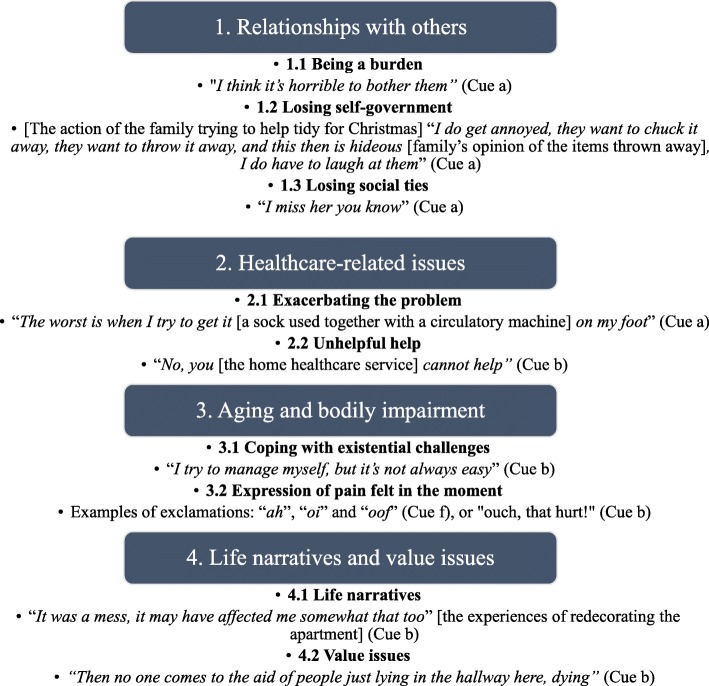
Thematic coding scheme for expressed worries with examples

The coding scheme was developed in a collaborative process between the authors led by the first author (LH). Several drafts of the coding manual were discussed, revised, and then finalized in consensus between the authors. All expressions in the data material could be assigned to one of the four mutually exclusive main themes, and to one of their mutually exclusive sub-themes (Fig. 1).

Figure 1 shows the coding scheme, including examples of actual patient expressions.

#### Inter-rater reliability of thematic coding scheme

Cohen’s kappa (κ) was calculated for a random sample of 78 cues/concerns (16 visits) coded by the first (LH) and second (VS) author independently. For the main themes, simple agreement between the coders was 86%. The inter-rater reliability, taking into account the possibility of the agreement by chance using Cohen’s kappa, was κ=0.68. For coding of sub-themes, simple agreement was 80% and inter-rater reliability was κ = 0.64. After establishing inter-rater reliability, the first author (LH) coded the remaining visits. Simple agreement and intra-rater reliability for the first author based 46 cues/concerns (16 visits) was 93% and κ = 0.91, respectively.

### Care provider responses

**Fig. 2:**
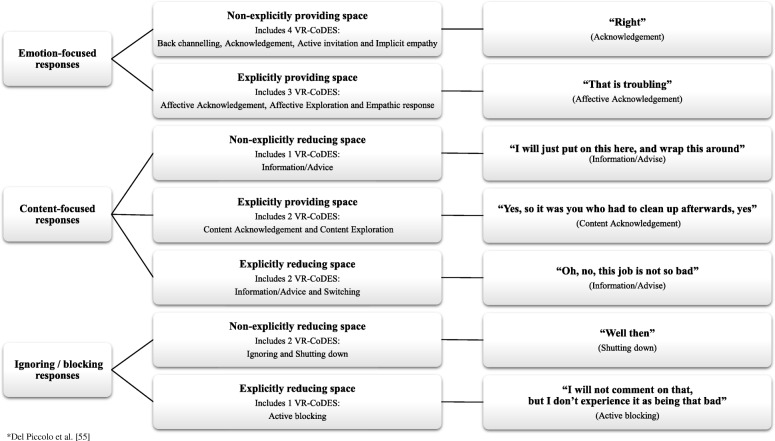
Adapted Provider response sum-categories

The response was assigned one of 15 eligible, mutually exclusive codes based on the two VR-CoDES dimensions [34, 54]: First, whether the response refers to the cue/concern *explicitly* or *non-explicitly,* that is whether or not the nursing staff response maintain wording or key elements of the concern/cue, which reflects the nursing staff’s ability to keep focused attention on the patient expression, hinting to the nursing staff’s interest for the patient’s way of expressing him/herself [55]. Second, the coding system address whether the response *provide space* or *reduce space* for further disclosure of the concern/cue, that is whether or not the response invites the patient to talk more about the expressed worries. This indicates if the response communicates an intention of maintaining reciprocity between patient and nursing staff, a change of topical focus, or introduce a complete topic shift [56]. Two VR-CoDES response codes were not coded; “non-explicitly providing space – silence” as it is not applicable to audio-recordings [54] and the code “explicitly reducing space – postponement” which was not identified in the material. The VR-CoDES-response codes were further grouped into three sum-categories based on communicative function: 1) Responses with a focus on the emotion, 2) Responses with a focus on content, and 3) Ignoring/blocking responses [41], Fig. 2. The three sum-categories allowed differentiation of response behaviours that: 1) acknowledge and/or elaborate the expressed emotions and encourage further disclosure of the affective dimension of the expression, 2) pay attention to the factual dimension of the expression, and 3) ignore or divert attention away from the expressed worry. This allows analysis of communicative function with regard to the thematic content of worries.

### Statistical analysis

Statistical analysis was performed with IBM SPSS Statistics, version 24.0 (IBM, Armonk, NY, USA). Missing data were only identified for nursing staff demographic data (Table 1). Frequency and summation statistics were used to describe the sample, themes and nursing staff responses. Differences in response behaviour between the professions (RN or NA) with regard to themes of worries were explored. Pearson’s chi-squared test or Fisher’s exact test were used to identify group differences relating to cues/concerns, themes of worries, nursing staff responses, and profession. The significance level was set at < 5%, and adjusted residuals (cut-off set at ≤–2 or ≥2) guided analysis of associations between categorical variables [51]. An adjusted residual exceeding ±2 for a given nursing staff response, indicated that the response was more or less likely to occur to a given theme of cues/concern than the other responses.

### Research ethics

All steps of the research process and the preparation of this paper were guided by the World Medical Association’s Declaration of Helsinki: Ethical principles for medical research involving human subjects [57]. All data handling was in accordance with Norwegian legislation and institutional policy. The Data Protection Official for Research approved the study (project number: 36017). The Research Council of Norway (PraksisVEL, grant no. 226537) and the University College of Southeast Norway, Faculty of Health and Social Sciences, Drammen funded the project. The funding sources played no part in the research.

## Results

The data eligible for analysis in this paper included 638 patient expressed worries (cues/concerns) and 641 subsequent nursing staff responses. Cues/concerns were identified in 74% (144/195) of the audio-recorded home care visits. A mean of 4 patient cues/concerns (SD ±4.2, range 1 to 25) were identified in visits with expressed worries. Worries were most frequently expressed using metaphors, emphasis, profanities, or expressing uncertainties and hope (VR-CoDES, cue b), followed by the use of vague or unspecified words describing the emotion (VR-CoDES, cue a), and clear and unambiguous expressions describing an unpleasant current or recent emotion (VR-CoDES, concern), Table 2. In addition, a small number of worries were emphasizing unpleasant cognitive or physical states (VR-CoDES, cue c), expressions standing out from the narrative (VR-CoDES, cue d), repetition of neutral words/phrases (VR-CoDES, cue e), and non-verbal hints of unpleasant emotions like crying or sighing (VR-CoDES, cue f).

**Table 2:** Elicitation and type of VR-CoDES – cues and concerns in relation to themes

Themes	Elicitation of worries		VR-CoDES – cues and concerns^a^				Total
	Nursing staff elicited	Patient elicited	Concern	Cue a	Cue b	Cue c-g	
Relationships with others (%)	37 (63)	22 (37)	8 (14)	20 (34)	30 (51)	1 (2)	59 (100)
Adjusted Residual	1,1	−1,1	0,9	3,6**	− 1,7	− 2,5**	
Health care-related issues (%)	64 (65)	34 (35)	7 (7)	16 (16)	67 (68)	8 (8)	98 (100)
Adjusted Residual	2,0*	− 2,0*	− 1,0	−0,2	1,6	−1,2	
Aging and bodily impairment (%)	212 (50)	209 (50)	40 (10)	61 (14)	277 (66)	43 (10)	421 (100)
Adjusted Residual	−4,1*	4,1*	− 0,3	−2,5**	3,4**	− 1,8	
Life narratives and value issues (%)	45 (75)	15 (25)	8 (13)	12 (20)	16 (27)	24 (40)	60 (100)
Adjusted Residual	3,1*	− 3,1*	0,9	0,7	− 5,8**	7,1**	
Total patient expressions of worries (%)	358 (56)	280 (44)	63 (10)	109 (17)	390 (61)	76 (12)	638 (100)

Percentages rounded to nearest whole percentage

*Pearson Chi-Square showed significant association between elicitation of worries and themes (*p*˂0,001). Adjusted residuals indicate association between themes and who elicit the expression

**Pearson Chi-Square showed significant association between type of patient expressions and themes (*p*˂0,001). Adjusted residuals indicate association between themes and how worries are expressed

^a^*Concern:* clear and unambiguous expressions describing an unpleasant current or recent emotion; *Cue a:* the use of vague or unspecified words describing the emotion; *Cue b:* hints about hidden concerns; *Cue c-g:* other type of hints to underlying negative emotion [25]

### Content of older persons’ worries

“Ageing and bodily impairment” was the most frequent theme of worries, including 66% (421/638) of all cues/concerns. Of these, 69% (290/421) captured worries about *Coping with existential challenges*, whereas 31% (131/421) were *Expression of pain felt in the moment*. Adjusted residuals show that worries about “Ageing and bodily impairment” were more frequently expressed as hints by use of emphasis, profanities, or expressions of uncertainties and hope (cue b) (Table 2).

Only 15% (98/638) of the expressed worries were associated with “Health care-related issues”. Worries addressing “Relationships with others” (9%; 59/638) and “Life narratives and value issues” (9%; 60/638) were least frequently expressed. Only two of the worries related to “Relationships with others” addressed losing self-government. Adjusted residuals showed that worries about “Relationships with others” were more likely expressed using vague or unspecified words for the unpleasant emotion (cue a), and “Life narratives and value issues” were more likely expressed using other type of hints to underlying unpleasant emotion like crying, repetitions or descriptions of unpleasant cognitive or physical states (cue c-g). “Health care-related issues” showed no difference in terms of how the worries were expressed. Table 2 shows who initiated the cues/concerns and how cues/concerns were expressed with regard to theme and VR-CoDES code. Figure 1 provides examples of patient expressions of worries for the themes and sub-themes.

### Elicitation of themes

In general, RNs and NAs most often elicited the expressions of worries. This was especially obvious for expressions related to “Life narratives and value issues” (Table 2). For worries about “Ageing and bodily impairment”, the majority of worries related to *Coping with existential challenges* were elicited by nursing staff, whereas *Expression of pain felt in the moment* was largely elicited by the patient (61% (177/291) versus 73% (95/130), Chi-square *p* < 0.001).

### Nursing staff responses

Overall, 47% of the responses focused on the emotional component of cues/concerns, followed by responses focusing on content (32%) and responses ignoring or blocking emotional expressions (21%), Table 3. Table 4 provides examples of dyads found in the material, illustrating different type of nursing staff responses used in relation to the main themes. How nursing staff responded was associated with theme of worries (Pearson Chi-Square, *p* = < 0,001). There was no significant difference in responses between NAs and RNs.

**Table 3:** Themes and sub-themes by response sum-categories

Themes	Sub-themes	Emotion-focused responses (Adjusted Residual)	Content-focused responses (Adjusted Residual)	Ignoring/blocking responses (Adjusted Residual)
Relationships with others	Being a burden	11 (−3,1)*	23 (2,8)*	5 (0,3)
	Losing self-government	1 (0,2)	1 (0,1)	0 (−0,5)
	Losing social ties	13 (3,1)*	3 (− 3,0)*	2 (− 0,1)
	Sum, n (%)*	*25 (42)*	*27 (46)*	*7 (12)*
Health care-related issues	Exacerbating the problem	22 (0,0)	20 (0,0)	8 (−0,1)
	Unhelpful help	21 (0,0)	19 (0,0)	8 (0,1)
	*Sum, n (%)*	*43 (44)*	*39 (40)*	*16 (16)*
Aging and bodily impairment	Coping with existential challenges^a^	149 (3,2)*	89 (0,3)	55 (− 4,0)*
	Expression of pain felt in the moment	45 (−3,2)*	38 (0-,3)	48 (4,0)*
	Sum, n (%)*	*194 (46)*	*127 (30)*	*103 (24)*
Life narratives and value issues	Life narratives	37 (1,0)	10 (−0,2)	4 (−1,3)
	Value issues	5 (−1,0)	2 (0,2)	2 (1,3)
	*Sum, n (%)*	*42 (70)*	*12 (20)*	*6 (10)*
Total responses^a^	*n* = 641 (100%)	*n* = 304 (47%)	*n* = 205 (32%)	*n* = 132 (21%)

Percentages rounded to nearest whole percentage. Sum scores in italic indicate the number (%) of response categories for each main theme

*Fisher’s Exact Test showed a significant association between response sum-categories and sub-themes within “Relationships with others” (*p* = 0,009) and “Aging and bodily impairment” (*p*<0,001), respectively. Adjusted residuals indicate the association between sub-themes and response behaviour

^a^Three expressions within *Existential challenges* were met by a double response leaving total number of responses as lager than total number of patient expressions of worries (Table 2)

**Table 4:** Dyads illustrating different combinations of main themes and type of responses^a^

Themes	Patient expression	Examples of responses assigned to the three sum-categories of responses	
Relationships with others	*“If it gets much, a lot, then the nerves kicks in you see”* [Talking about a family member]	*“Yes that’s …”* [Pauses to allow the patient to continue]	*Emotion-focused responses*
	*“Poor thing, yes, she is very clever, but I do feel sorry for them, having to work on this”* [Worries that the nursing staff thinks caring for her wound is not a nice task, focus is on the relation not the care delivery]	*“It is our job you know”* [Provides information/advice]	*Content-focused responses*
	*“Oh, well, a lot of stuff, you have to deal with a lot of strange stuff because of me”* [Patient is getting dressed, focus is on the relation not the care delivery]	[Nursing staff do not comment, focus on next task]	*Ignoring / blocking responses*
Health care-related issues	*“You see, there is no point telling the doctor about this, I don’t get any answers anyway”* [Discussing need for medical care]	*“No?”* [Tone of voice invite the patient to elaborate]	*Emotion-focused responses*
	*“Everything is just yuck”* [The nursing staff has first suggested that the patient needs to eat more]	*“No, but it is just so that if you do, you don’t have to get so tired because like it is, you don’t get much nutrition, and then you have no energy”* [Provides information/advice]	*Content-focused responses*
	*“I do feel the male staff is the worst”* [Is not happy with some of the staff at a care service institution]	*“Yes, no, I won’t comment on that. But I don’t see it like that.”* [Shuts down further discussion, the topic changes]	*Ignoring / blocking responses*
Aging and bodily impairment	“*I try to manage myself, but it’s not always easy”* [The patient shares her thoughts on being dependant of home care because of bodily impairment]	*“No”* [Tone of voice invite the patient to elaborate]	*Emotion-focused responses*
	*“It’s this damn knee!”* [The patient spontaneously raises this topic]	*“Do you have pain in your knee?”* [The nursing staff asks specifically about the content and maintains wording used by patient]	*Content-focused responses*
	*“And I understand, I am not feeling very fit these days you see”* [The patient talks about trying to cope with reduced function]	*“No, but you’re fine. You just need to take your time moving about.”* [Shuts down further discussion, the topic changes]	*Ignoring / blocking responses*
Life narratives and value issues	*“No, that was …”* [Shares a story about experiencing a loss in the family]	*“mm?”* [Tone of voice invite the patient to elaborate]	*Emotion-focused responses*
	*“Very sad that it always has to hit the best of us”* [Shares a story about experiencing a loss in the family]	*“Yes, many people experiences this”* [Provides general information]	*Content-focused responses*
	*“Yes, it is, but when we …”* [Starts telling about traditions from younger days, but gets interrupted]	*“Yes, now a days it is a lot of pressure”* [Shuts down further discussion, the topic changes]	*Ignoring / blocking responses*

^a^Phrasing used in the dyads have been altered when deemed necessary to ensure confidentiality e.g. nursing staff is used instead of their name and care service is used instead of the name of the institution

#### Responding to different sub-themes within “Ageing and bodily impairment”

Nursing staff responded differently to “Coping with existential challenges” and “Expression of pain felt in the moment”. “Coping with existential challenges” received more frequently responses focussing on the expressed emotion than responses ignoring/blocking the expression of worry (Adjusted residuals 3.2 versus − 4.0, Fisher’s Exact Test, *p*<0,001). Responses to “Expressions of pain felt in the moment” were more frequently ignored/ blocked than responded to with focus on the expressed emotion (Adjusted residuals 4.0 versus − 3.2, Fisher’s Exact Test, *p*<0,001), Table 3.

#### Responding to different sub-themes within “Relationships with others”

Worries about “Being a burden” was relatively more often responded to with a content-focused response than with an emotion-focused response (Adjusted residuals 2.8 versus − 3.1, Fisher’s Exact Test, *p* = 0,009), whereas worries about “Loosing social ties” were relatively more often responded to with an emotion-focused response than with a content-focused response (Adjusted residuals 3.1 versus − 3.0, Fisher’s Exact Test, *p* = 0,009), Table 3.

#### Responding to “Healthcare related issues” and “Life Narratives and value issues”

There were no significant differences between types of responses for sub-themes within “Healthcare related issues” and “Life narratives and value issues”, Table 3. Of the responses addressing “Healthcare related issues”, 44% were responding to the emotion, 40% were responding to the content, and 16% were ignoring/blocking emotional expressions. Responses to “Life narratives and value issues” were 70% emotion-focused, 20% content-focused and 10% ignored or blocked the expressed worries.

## Discussion and conclusion

### Discussion

To the best of our knowledge, this is the first study to explore the thematic content of patient expressed worries in home care visits, and how the nursing staff respond to different thematic content. The findings show that the thematic content to a large extent focus on “Ageing and bodily impairment”, that is the older persons’ personal struggle to cope with an aging body, reduced physical health and pain, and to accept that life is near the end [18]. Nursing staff response differ according with thematic content of the older persons’ worries, especially for older persons’ worries about “Relationships with others” and “Aging and bodily impairment”.

“Ageing and bodily impairment” represent existential challenges because the addressed circumstance or event challenges the individual person’s identity, experience of autonomy, self-worth and/or dignity [58]. Reduced function and chronic pain may leave the person unable to engage in valued activities and force the person in to a state of enduring pain, which hampers the prospect of living a good life. This is shown as a strong trigger for existential challenges which can lead to death anxiety [59], reflecting the importance of supportive nursing staff responses to such worries [60, 61].

Moreover, bodily impairment may influence the older person’s ability to communicate properly, and act as a barrier for communication, and nursing staff may experience the older person’s bodily impairment as an obstruction to communication, and a barrier to respond effectively to expressions of existential loneliness among older persons [62]. The nursing staff’s lack of confidence in own ability to correctly interpret the older persons’ care needs in these situations hamper their ability to provide effective care [62]. An international task force of experts have emphasised the importance of implementation strategies to prevent functional decline in older persons living in nursing homes to ensure quality and efficient care for older persons living in the community [63]. Loss of mobility and social support, cognitive decline, iatrogenic events, and progression of disease are described by the task force as driving factors of functional decline among older persons. These are all issues expressed as causing worry by the older persons in this study. This highlights the importance of ensuring that these issues are taken into consideration when the nursing staff evaluate home care needs of older persons. Moreover, a clinical practice that includes the older person’s perspective and worries about functional decline and bodily impairment is likely efficient in ensuring the older person’s ability to continue to live a good life in their own home and has potential to reduce or postpone the need for transferal to a nursing home. There is some evidence indicating that investments in home care services may influence the need for nursing homes, although the evidence is weak and has methodological challenges [64].

In this study, the older person’s effort to cope with functional decline also raises existential issues for the person and is captured by the main theme “Ageing and bodily impairment”. Because of the high frequency of worries about existential issues in home care, there is a need for communication training of nursing staff to ensure sensitivity to these worries, and to provide nursing staff with adequate strategies to provide support. What type of responses older persons prefer and perceive as supportive in different situations are yet to be explored. However, increased communication skills may have the potential to enhance the experienced quality of care [7, 8], prevent unnecessary suffering [65] and increase the nursing staff’s perception of being competent when facing challenging communication in home care [26, 27, 62].

In general, the older persons in our study spontaneously expressed a greater number of worries compared to patients in other care situations in previous studies [35, 36, 38]. This was especially prominent for the theme “Ageing and bodily impairment”, which had equal distribution of nursing staff- and patient-elicited cues/concerns.

The fact that nursing staff were more prone to elicit cues/concerns for other themes of worries than “Ageing and bodily impairment” may be linked to features relating to the older person, the nursing staff, or the theme itself. One interpretation could be that older persons are more inclined to share worries about “Ageing and bodily impairment”. Another interpretation could be that nursing staff focus less on issues relating to ageing and bodily impairment, leading older persons to initiate such worries by including the topics into the conversation. Finally, this could be related to nursing staff finding it difficult to be supportive and helpful when facing such worries, because they tap into existential issues and need for spiritual support, an area where nursing staff lack competence [26, 66, 67]. Consequently, nursing staff may feel uneasy and wait for the older person to include these topics into the conversation rather than introducing them.

When exploring responses used by nursing staff in relation to worries about “Ageing and bodily impairment”, our findings revealed that existential challenges were responded to with an emotional focus and thereby inviting the older person to talk more about the topic, whereas expressions about pain were more likely to be ignored. Hence, the nursing staff in our study seem to be attentive when the expressions include a reference to existential challenges, but avoiding potentially challenging situations [26] by ignoring the patient's expressions of pain. This may indicate that nursing staff do recognise the older person’s need to share his/her worries about existential issues and respond in a way that allows the older person to talk more about the topic. This supports other research that emphasises that nursing staff need strategies for dealing with existential issues in a more profound way than simply allowing the person to express him/herself [62, 66].

When the nursing staff ignore the spontaneous expressions of pain, this may not be a conscious response strategy, but rather an indication of the nursing staff becoming less responsive or even disinterested over time, with regard to patients known to be expressive about their pain [50, 68]. In this way, they may not reflect on whether this could be the older person’s way of hinting to how the pain is emotionally challenging or overwhelming, but rather interpret this as “normal” for the situation at hand. Addressing psychosocial aspects of living with chronic pain have been emphasised as salient and often neglected by care providers [69].

Inadequate pain management may lead to adverse physical and psychological patient outcomes, like reduced wound healing, reduced immune system response, negative effects on vital bodily functions and reduced patient mobility [70]. Nursing staff sensitivity towards the patient’s experience of pain and efficient pain management may consequently prevent or reduce functional decline from poor pain management. This has been emphasised as essential for practicing efficient geriatric medicine and care in the setting of nursing homes [63], and is likely equally important in home care for older persons.

Given the assumption that a person’s experience of pain can challenge a person’s emotional well-being, the nursing staff’s evaluation of and attention to expressed worries during home care delivery will likely influence their ability to tailor the care in accordance to person-centred principles, like practicing holistic care and eliciting the patient perspective [11]. Ensuring high quality pain management as part of home care services includes tailored care for the emotional aspects of living and coping with pain, and the influence of daily life of the older person. Evidence support that home care nursing staff and their patients develop a relationship that allow home care nursing staff detailed insight into the personal life of the patient, and therefore a more comprehensive picture of the person’s life story than in a hospital setting [30]. This highlights a need for collaboration between nursing staff, who provide the daily care and observe how pain and bodily impairment impact the older person’s life, and the general practitioners and other healthcare services, to provide a holistic approach at all levels of care delivery. This is promoted as essential to provide person-centred care for older persons [15].

Both existential challenges and pain management capture worries that involve older persons’ perceived self-determination, functional status, and effort to maintain health [18]. These elements are highlighted as essential to ensure the ability of older persons to continue a good life living at home, and are important care outcomes for home care services [71–75]. Our study supports recommendations made by other studies [26, 76], that existential challenges and pain management should be emphasized in communication skills training for students and nursing staff working in home health care. Moreover, our study indicates that addressing how to cope with chronic pain and reduced functional ability caused by pain, may be important to include in communication training of nursing staff together with existential issues like the influence of an aging body on a person’s identity. How to remain sensitive and responsive may be particularly important in relation to these issues, including the nursing staff’s ability to listen [68] and to respond in a supportive way [41], which both are essential components in person-centred quality care [6, 12].

“Relationships with others”, and “Life narratives and value issues”, which address psychosocial aspects [18], were the least frequently expressed worries in our study. The findings were somewhat surprising to the authors. Both themes are assumed to be relevant for the experience of isolation and loneliness [18], thereby potentially influencing health and well-being of older persons [77, 78], and seen as important challenges to manage in care of older persons [17]. Therefore, we expected these themes to be frequently observed. One reason for our findings could be that the care setting is characterised by task focused communication [27] that places the care tasks and related issues in focus. Therefore, the patients may by nature experience the company of the nursing staff as supportive and thereby drawn the attention away from potentially worrying relational issues or important life events.

This study also found that older persons’ worries about “Relationships with others” were responded to differently depending on the sub-theme. The sub-theme “Being a burden” received a higher proportion of content-focused responses, while the sub-theme “Losing social ties” received more emotion-focused responses. This may indicate that nursing staff perceives responding to the emotion as the best approach to worries about losing significant others, allowing the older persons to explore their feelings using their own words and following their own train of thoughts. The experience of being a burden may be seen as best addressed by directing the older person’s attention the factual aspects of the situation. Both type of responses indicate that the act of whole-heartedly listening to the patient is essential because it facilitates the person’s perspective and fosters the care relationship [68].

Responding to content when addressing issues relating to being a burden to others, may represent how nursing staff perceive best practice as providing information, reasoning or solutions, by presenting facts or understandings, helping the older person to gain perspective on the situation or circumstance triggering the unpleasant emotion. This has been argued as a more effective approach in clinical communication when trying to help patients to regulate worry as compared to focusing on the affective component of the patient’s expression [79]. However, the type of responses focusing on thematic content is perceived as only moderately supportive and most effective when combined with statements that also acknowledge the affective component of the person’s worry [65]. Given the unresolved issues relating to when and if an affective focus is preferred by patients and when emotion-focused responses may contribute to more effective care delivery, further research need to explore cause and effect on outcomes relating to patient preferences, patient health, and the care relationship.

For “Life narratives and value issues”, expressions of worries facilitated by nursing staff was particularly prominent. This theme was also dominated by responses that allowed the patient to talk more about their worries. Evidence supports the importance of nursing staff engaging in older persons storytelling to allow shared understanding about their life-story, and to foster rapport and deepen the care relationship [80]. This emphasises that nursing staff working in home care should continue to be sensitive to the older person’s need to share narratives and important experiences from their own life.

Only two expressions were related to worries about “Losing self-government”. Moreover, a relatively small proportion of expressions concerned “Health-care-related issues”. Therefore, it appears that older persons rarely express worries regarding help being experienced as invading or insensitive to their preferences. Other studies have described the importance of detecting and disclosing such feelings in order to provide efficient care and to succeed in helping older persons to live a good life in their own homes [81, 82]. One reason for this finding may be that older persons may have difficulty in expressing dissatisfaction with the care provided and how the help is organized by the nursing staff. In turn, such reluctance may hamper the nursing staff’s ability to provide person-centred care, because the individual preferences, experiences, and ideas about what care needs are not shared by the patient [83]. Another explanation could be that nursing staff is already responsive and sensitive, and therefore care provision is rarely an issue causing worry. Based on these findings, it is important to examine further how older persons are included in care management and decisions on a daily basis, how to best involve them, and nursing staff strategies for doing so.

Emotion-focused responses, allowing the older person to elaborate on worries may serve to build the relationship through sharing of experiences. The nursing staff show respect for the older person by actively listening and allowing a shared understanding of events that may influence how the older person understands and sees him/herself at the present time. From this perspective, emotion-focused responses may be a way of practising person-centred communication [82, 84]. However, this depends on whether or not the nursing staff actively use the information to tailor the care in accordance with the perspectives raised by the older person. Limiting the engagement with the older person’s worries to allowing him/her to talk about these issues, deny the important function of offering new perspectives that can help the older person to cope better with the situation and regulate the experience of worries [85]. A person’s subjective experience of important circumstances or conditions in life, such as relational issues or threats to personal health and well-being, may all cause worries and distressful thoughts leading to the need to share, discuss, and seek comfort and advice in others [85]. Evidence suggests that older persons’ perceived well-being and life satisfaction are positively correlated with perceived good health, absence of worry and self-esteem [86]. This suggests that supportive responses—providing explicitly relevant information to aid understanding, offering a solution to a problem, providing comfort or emotional support, or helping the older person to explore what is experienced as worrying [65, 85] may be important to facilitate a general and sustained feeling of well-being.

Although there are uncertainties about the effects of engaging with patients’ emotional cues [87], responsiveness to emotional expressions is emphasised as important to manage communication challenges and to prevent unnecessary suffering for the patient [26, 35, 76]. Recognising and responding to emotions tap into preferences and values of the individual patient [82, 88], and potentially strengthen the care relationship [84, 89].

### Strengths and limitations

The relatively large study sample strengthen the findings of the study, especially because the study sample had features related to care assignment, nursing staff and patients that were similar to those described as typical of home care visits [71, 73, 74]. The assumption that audio-recorded data is a valid source to use in communication ratings is supported by other studies, communication ratings using audio and video highly correlate [90], and audio-recording seems to have little influence on the actual communication unfolding between the care provider and the patient [91]. Nevertheless, given that knowledge about emotional talk between older persons and nursing staff in home care is scarce, there is a chance that vital variables have been unknowingly omitted.

The coding scheme focusing on the thematic content in older persons’ expressed worries builds on findings from qualitative in-depth analysis of emotional talk unfolding in care situations extracted from the study sample [18]. This empirically driven development may have contributed to strengthening the validity of the coding scheme and ensuring relevance for the setting being analysed [92]. This is further supported by how all cues/concerns could be assigned to a particular theme and related sub-theme, and the substantial agreement between coders [93]. The transferability of the coding scheme can only be evaluated though conducting studies that apply the coding scheme to samples that are representative of other care settings and populations.

The coding rule given by VR-CoDES to assign all periods of silence the code non-explicitly reducing space by ignoring the cue or concern [54] may be a source of bias. We know that silence is a communication strategy found to elicit concerns [94]. Hence, this way of coding may overestimate the number of responses “non-explicitly ignoring” patients’ worries, painting a picture of nursing staff as more inclined to reduce space for further emotional disclosure than actually being the case. However, to code a pause as “silence” requires a period of minimum three seconds of no talk [54]. This was very rare in this study and hence unlikely to influence the results.

### Conclusion

The novel coding scheme, designed to differentiate thematic the content of older persons’ worries captured by VR-CoDES, allows subsequent nursing staff’s responses to be analysed with regard to thematic content of worries. Worries about “Ageing and bodily impairment” are most frequently expressed, and spontaneously elicited by the older person in home care. Nursing staff address emotions when the expressed worries regard existential challenges, fear of losing social ties, or captured life narratives and value issues important for the older person. Nursing staff focus on the content when responding to the experience of being a burden and are more likely to ignore spontaneously expressed pain. Further research exploring the influence of nursing staff’s use of different type of responses on quality of care and patient satisfaction is needed. Research investigating how nursing staff address and tailor care towards existential issues, including coping with changes caused by the aging and living with pain, seems salient to ensure quality care and person-centred home care delivery. Our findings show that characteristics of thematic content of patients’ worries and nursing staff’s responses are associated, and is likely valuable to include in communication training for students and nursing staff.

## Abbreviation

ADL: Activity of daily living
COMHOME: Person-centred communication with older people receiving healthcare
NA: Nurse Assistant
RN: Registered Nurse
VR-CoDES: Verona coding definitions of emotional sequences
